# Current practice of orthopaedic surgical skills training raises performance of supervised residents in total knee arthroplasty to levels equal to those of orthopaedic surgeons

**DOI:** 10.1007/s40037-018-0408-y

**Published:** 2018-02-23

**Authors:** Luuk Theelen, Cheryll Bischoff, Bernd Grimm, Ide C. Heyligers

**Affiliations:** 1 Department of Orthopaedic Surgery, Zuyderland MC, Heerlen, The Netherlands; 2 AHORSE Research Foundation, Department of Orthopaedic Surgery, Zuyderland MC, Heerlen, The Netherlands; 3 School of Health Professions Education, Maastricht University, Maastricht, The Netherlands

**Keywords:** Total knee arthroplasty, Orthopaedic surgery, Orthopaedic residency, Surgical training, Training hospital

## Abstract

**Aim:**

To investigate whether the current, generally accepted practice of orthopaedic surgical skills training can raise the performance of supervised residents to levels equal to those of experienced orthopaedic surgeons when it comes to clinical outcomes or implant position after total knee arthroplasty.

**Methods:**

In a retrospective analysis of primary total knee arthroplasty outcomes (minimum follow-up of 12 months) procedures were split into two groups: supervised orthopaedic residents as first surgeon (group R), and experienced senior orthopaedic surgeons as first surgeon (group S). Outcome data that were compared 1 year postoperatively were operation times, complications, revisions, Knee Society Scores (KSS) and radiological implant positions.

**Results:**

Of 642 included procedures, 220 were assigned to group R and 422 to group S. No statistically significant differences between the two groups were found in patient demographics. Operation time differed significantly (group R: 81.3 min vs. group S: 71.3 min (*p* = 0.000)). No statistically significant differences were found for complications (*p* = 0.659), revision rate (*p* = 0.722), femoral angle (*p* = 0.871), tibial angle (*p* = 0.804), femoral slope (*p* = 0.779), tibial slope (*p* = 0.765) and KSS (*p* = 0.148).

**Discussion and conclusion:**

Supervised residents needed 10 minutes extra operation time, but they provided the same quality of care in primary total knee arthroplasty as experienced orthopaedic surgeons concerning complication rates, revisions, implant position on radiographs and KSS. The currently used training procedure in which the supervising surgeon and the resident decide if the resident is ready to be first surgeon is safe for patients.

## What this paper adds

In a retrospective study we analyzed the outcomes of primary total knee arthroplasties performed by supervised orthopaedic residents and senior orthopaedic surgeons. Only small differences were found regarding operation time, but no significant differences in other outcomes such as complication rates, radiographic position of the implant or patient-reported outcomes. Our study shows that the generally accepted postgraduate surgical training practice of surgery performed by supervised residents is safe for patients and that it raises the performance of supervised residents to levels equal to those of senior orthopaedic surgeons.

## Introduction

For several decades now, residents are trained in surgical skills in a master-apprentice relationship. This generally accepted practice in postgraduate medical training implies that training is integrated with patient care. This integration has several advantages, such as efficiency and exposure to actual clinical practice. Learning by doing has been proved to be an important learning tool [1]. In the Netherlands, orthopaedic training consists of an 18-month residency in general surgery followed by 4.5 years of residency in orthopaedic surgery. An important aspect of postgraduate medical training in orthopaedic surgery is acquiring surgical skills in total joint surgery, especially total hip and total knee arthroplasty. These skills are obtained gradually and stepwise. At first, residents observe surgical procedures and train *in vitro*, in a simulated situation using sawbones or in the anatomy lab [2]. This is to learn the different steps of the procedure and the tools that are used. In the next stage they perform increasingly complex tasks in assisting total hip and knee arthroplasties that are performed by experienced orthopaedic surgeons. They start to learn how the patient needs to be positioned on the operating table and the different stages to get an optimal surgical approach to perform the procedure. Then they perform different steps with increasing difficulty: preparation of the different bones of the hip and knee joint, and fixation of the implant parts of a total hip and a total knee prosthesis. When, after enough experience, the supervisor and the resident both consider the resident’s knowledge and skills to be at the desired level, the resident performs all the steps of the complete surgical procedure, assisted by the supervising surgeon. This moment is crucial because it is the first time that the resident is the leading surgeon. In the next stage, when the supervisors and the resident agree that this is safe, based on different supervised procedures, the complete procedure can be entrusted to the resident without direct supervision. In this way the entrustable professional activity (EPA) concept is applied in the training of orthopaedic residents [3].

While the training of residents during surgical procedures should not negatively influence patient safety, literature shows that more surgical experience is associated with less mortality, shorter length of hospital stay, and better implant position [4–6]. Resident involvement in laparoscopic appendectomies and inguinal hernia repairs showed a significantly longer operation time for residents than for experienced surgeons [7, 8], but no influence on complication rates [9]. One cohort study showed lower complication rates in case of resident involvement [10]. On the other hand, large cohort studies on joint arthroplasties found significantly higher complication rates and longer operation times when residents performed the procedure compared with experienced orthopaedic surgeons [11, 12].

Unfortunately, in these studies, the exact role of the residents and their supervisors remained unclear, and no sub-analyses were performed on hip or knee procedures. Besides, in these studies, no clear distinction was made between supervised residents and experienced orthopaedic surgeons as first surgeon. We wanted to know whether the training of surgical skills of orthopaedic residents poses a risk to the quality of patient care. Therefore, our aim was to investigate whether the current, generally accepted practice of orthopaedic surgical skills training can raise the performance of supervised residents to levels equal to those of experienced orthopaedic surgeons when it comes to clinical outcomes or implant position after total knee arthroplasty.

To investigate this, we compared the outcomes of total knee arthroplasties performed by supervised residents as leading surgeon or experienced orthopaedic surgeons 1 year after the procedure had been performed. In particular we compared outcomes that are of importance for patient care: operation time, complication rate, revision rate, functional results and radiological results. We hypothesized that postgraduate medical training does not lead to differences in clinical outcome or implant position 1 year after total knee arthroplasty performed by supervised residents or experienced orthopaedic surgeons. To our knowledge, this is the first study comparing the outcomes of total knee arthroplasties performed by supervised residents and senior surgeons in the daily practice of postgraduate medical training in such a detailed way. The results of this study can be used to improve postgraduate surgical skill training of orthopaedic residents.

## Methods

### Patient selection

We retrospectively analyzed outcome data of patients who had undergone a knee arthroplasty procedure in the Zuyderland Hospital, a regional teaching hospital in the south of the Netherlands, between January 2010 and June 2014. Patients with a primary total knee arthroplasty (Vanquard, Biomet, USA) were included if follow-up data were available for at least 1 year after surgery, including measurements of the position of the implants on radiographs and data on the Knee Society Scores (KSS). Patients who underwent a knee arthroplasty procedure were excluded if data were not complete, in case of revision arthroplasty, unicompartmental arthroplasty or only a patellofemoral arthroplasty, and if the procedure was performed with patient-specific instruments (Signature total knee arthroplasty, Biomet, USA).

### Design

Operation reports of the included patients were consulted to establish who had been the first surgeon. Patients were assigned to group R if the first surgeon was a resident supervised by a senior specialist, and they were assigned to group S if the first surgeon was a senior orthopaedic surgeon.

All patients had been evaluated with the KSS 1 year postoperatively; the patients were invited to visit the hospital for an X‑ray and to meet a trained investigator who administered the KSS questionnaire. The KSS is a validated outcome score for knee surgery. It is completed partially by the patient and partially by the physician. A score between 80 and 100 is excellent, 70 to 79 is good, 60 to 69 is fair and a score below 60 is poor [13].

**Fig. 1:**
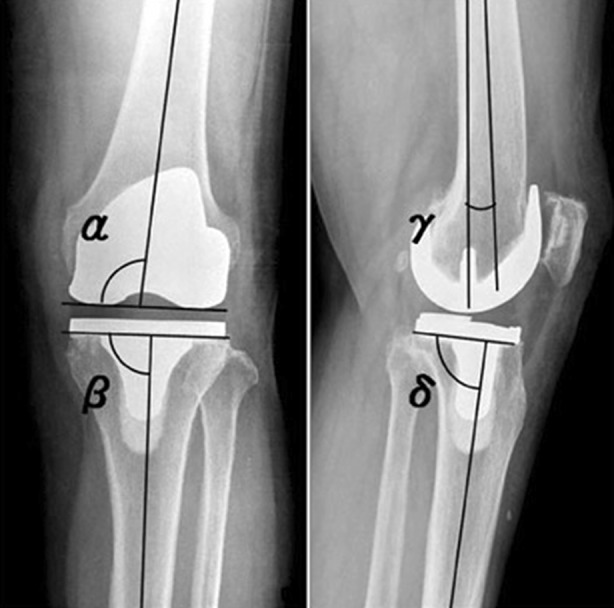
Femoral angle (α) and slope (γ), and tibial angle (β) and slope (δ) as measured on an anterior-posterior radiograph and on a lateral radiograph

Anterior-posterior and lateral radiographs taken 1 year postoperatively were analyzed by one investigator to evaluate the radiological position of the total knee arthroplasty, by measuring the femoral angle (α) and slope (γ) and the tibial angle (β) and slope (δ) as published previously (Fig. 1; [14]). Complication rates were evaluated based on official registration in patient records, with special interest for deep infections since these are most devastating to patients.

### Statistical analysis

Data included demographic patient information (age, gender, body mass index (BMI), American Society of Anesthesiologists (ASA) classification), information about the operation (duration in minutes, blood loss, implantation of patella component), and follow-up data (complications, revision surgeries, KSS, radiographic measurements). The patient demographics were of interest to determine whether there was a selection bias of potentially easier cases being operated by the residents, for example patients with younger age, lower BMI or lower ASA classification. The implantation of a patella component was relevant because this increases the operation time.

Data were analyzed with the independent samples T‑test and the Pearson Chi square test. Statistical significance was set at *p* < 0.05. All data were analyzed with SPSS (IBM Statistics version 24).

## Results

### Patient demographics

**Fig. 2:**
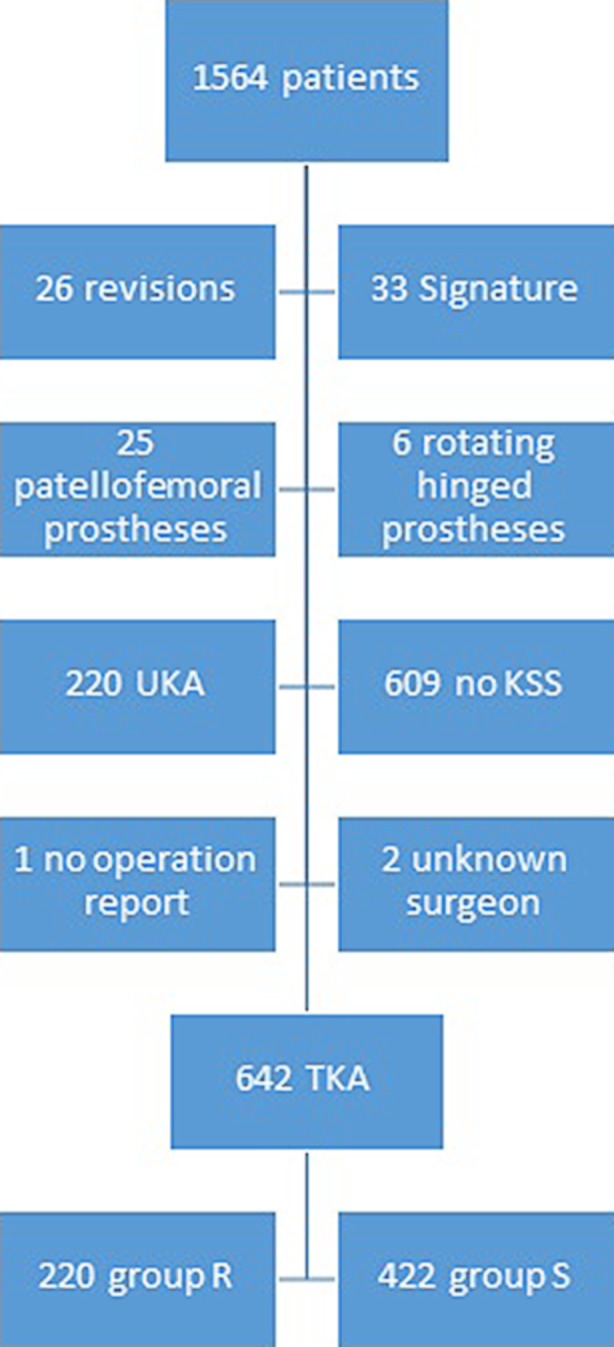
Flowchart of the patient selection process. (Signature is a technique that involves the use of patient specific instruments. *UKA* unicompartmental knee arthroplasty, *KSS* Knee Society Scores, *TKA* total knee arthroplasty)

Of the 1,564 patients who had undergone a knee arthroplasty in the Zuyderland Hospital between January 2010 and June 2014, 922 were excluded because of incomplete data, patient-specific instrumentations, revision arthroplasty, unicompartmental arthroplasty or patellofemoral arthroplasty. The remaining 642 patients were included in the study. Of these patients, 220 were operated by a supervised resident (group R) and 422 by an experienced orthopaedic surgeon (group S). The minimum follow-up time was 1 year, the mean follow-up time was 54 months. The flowchart of the patient selection process is shown in Fig. 2.

No statistically significant differences were found between both groups concerning the patient demographics age, gender, BMI, ASA classification and patella implantation (Tab. 1).

**Table 1:** Patient demographics

Patient demographics	Resident	Surgeon	*P* value
Age (years) mean	70.1	69.1	0.188
*Gender:*			0.797
Male (%)	36	38	
Female (%)	64	62	
BMI (kg/m^2^), mean	30.0	30.0	0.853
*ASA classification (%):*			0.654
ASA 1	10	10	
ASA 2	65	68	
ASA 3	25	22	
ASA 4	0	0	
Patella component (%)	67	68	0.820

*BMI* body mass index, *ASA* American Society of Anesthesiology

### Operation time and KSS

There was a statistically significant difference in operation time, 81.3 minutes in group R vs. 71.3 minutes in group S (*p* < 0.001). The KSS was 75.5 (46.3–104.6) for total knee arthroplasties performed by residents and 77.5 (47.1–107.4) for total knee arthroplasties performed by orthopaedic surgeons (*p* = 0.148) (Tab. 2).

**Table 2:** Operation time and Knee Society Score 1 year postoperatively

Variable	Resident (95% CI)	Surgeon (95% CI)	*P* value
Operation time, minutes	81.26 (47.9–115.3)	71.3 (31.4–111.2)	<0.001
KSS, 1 year postoperatively	75.5 (46.3–104.6)	77.5 (47.1–107.4)	0.148

### Radiological position

No differences were found between the groups concerning the position of the implants on radiographs (Tab. 3). The normal values for femoral angle are 92–97°, 90° for tibial angle, 0–3° for femoral slope and 83–90° for tibial slope [14].

**Table 3:** Radiological position of the implants

Variable	Resident (95% CI)	Surgeon (95% CI)	*P* value
Tibial angle	88.4 (84.5–92.4)	88.5 (84.3–92.7)	0.80
Femoral angle	94.5 (85.8–103.2)	94.4 (85.6–102.9)	0.87
Tibial slope	86.6 (79.9–93.4)	86.6 (79.6–93.6)	0.77
Femoral slope	−3.2 (−10.7–4.3)	−3.1 (−11.2–5.1)	0.78

### Complications

Complications did not differ significantly between the two groups (Tab. 4). In group R, 21 complications occurred, versus 35 in group S (*p* = 0.659). One deep infection was found in group R and four were found in group S (*p* = 0.665).

**Table 4:** Numbers and percentages of complications

Variable	Resident (*n* = 220)	Surgeon (*n* = 422)	*P* value
Hypovolaemic shock	1 (0.45%)	0 (0.00%)	0.343
Hypotension	3 (1.36%)	1 (0.24%)	0.119
Cardiac arrhythmia	1 (0.45%)	0 (0.00%)	0.343
Pulmonary embolism	0 (0.00%)	1 (0.24%)	1.000
Deep vein thrombosis	3 (1.36%)	1 (0.24%)	0.119
Superficial surgical site infection	3 (1.36%)	7 (1.66%)	0.774
Deep surgical site infection	1 (0.45%)	4 (0.95%)	0.500
Wound defect	1 (0.45%)	1 (0.24%)	1.000
Erysipelas	1 (0.45%)	0 (0.00%)	0.343
Blistering	0 (0.00%)	3 (0.71%)	0.555
Patella dislocation	0 (0.00%)	2 (0.48%)	0.549
Loosening	1 (0.45%)	2 (0.48%)	1.000
Disability in flexion/extension	5 (2.27%)	4 (0.95%)	0.287
Peripheral nerve lesion	1 (0.45%)	2 (0.47%)	1.000
*Total*	*21 (9.55%)*	*33 (7.82%)*	*0.651*

### Revisions

Revisions did not differ significantly between the two groups. Two revisions occurred in group R and six in group S (*p* = 0.722). The reasons for revisions are listed in Tab. 5.

**Table 5:** Reasons for revision

Revision	Reason	Surgery
*Group R*		
Patient 1	Aseptic loosening tibia component	Revision TKA
Patient 2	Instability	Change of insert
*Group S*		
Patient 1	Patellofemoral complaints	Patella component
Patient 2	Loosening locking bar	Revision of locking bar
Patient 3	Patella dislocation, rotation of femur component	Revision femur component, placement patella component, lateral release
Patient 4	Deep infection	Two-stage revision
Patient 5	Aseptic loosening tibia component	Revision tibia component
Patient 6	Flexion and extension impairment	Revision of insert

## Discussion

The aim of this study was to investigate whether the current, generally accepted practice of orthopaedic surgical skills training can raise the performance of supervised residents, who are considered ready to perform this procedure as first surgeon, to levels equal to those of experienced orthopaedic surgeons when it comes to clinical outcomes or implant position after total knee arthroplasty. In generally accepted practice the resident’s proven knowledge and skills and the conviction of the resident and the supervising surgeon are the base for entrusting the resident to perform the operation without a negative influence on patient outcome. This is also the aim of the concept of entrustable professional activities [3]. To establish whether the widely used procedure in postgraduate surgical skills training is justified in case of total knee arthroplasty, this study compared the outcomes at 1‑year follow-up for total knee arthroplasties performed by supervised residents on the one hand and by senior orthopaedic surgeons on the other. The results showed that patient demographics were the same in both groups, indicating that there was no bias towards easier cases being assigned to residents. Supervised residents needed 10 minutes extra operation time, but there were no significant differences in the outcomes that pose a risk to patient care: complications, revisions, KSS and radiological implant positions.

The radiological measurements were largely consistent with measurements of studies on the use of computer-assisted instrumentation in total knee replacement [15]. The position of the implants on radiographs did not differ between the two groups and were in line with the normal values.

Our finding that the operation time was 10 minutes longer in operations performed by supervised residents can be explained by lack of experience and corresponds with previous research in general surgery.[7, 8]. The difference in surgery time had no influence on complications, revision rate, implant position and KSS 1 year after surgery. Longer surgical procedures have the risk of more blood loss and more infections; however, the difference of 10 minutes on a total operation time of 80 or 70 surgery minutes is below the boundary [16].

The finding that there were no differences in patient outcomes other than operation time is not in line with previous studies that showed slightly higher complication rates in surgical procedures in which residents were involved. These studies, however, did not precisely describe the supervision and the activity of the residents [9–11]. Our findings show good results of the integration of resident training and patient care concerning primary total knee replacement as performed in an orthopaedic department of a training hospital. The deliberation between the resident and the supervising surgeon to decide if the resident is ready to perform the surgery under supervision is a safe procedure.

### Strengths and limitations

A strength of this study is that it focused on an authentic situation: the surgeons and residents who performed the operations were not aware of this study at the time of surgery. Besides, the patient data used in the present study were derived from various studies on follow-up outcomes after primary total knee arthroplasty that did not take the composition of the surgical team into account. Hence the investigators of those studies were not informed about who the first surgeon was, which means that they were effectually blinded for this parameter. Retrospectively, the current study related the outcome parameters to the information whether the first surgeon was an orthopaedic surgeon or a resident supervised by an orthopaedic surgeon.

There were several notable limitations to this study. First of all, it was a retrospective cohort study which might have implied a selection bias towards easier cases in the resident group. Our data, however, did not show significant differences in patient characteristics between the two groups. Secondly, follow-up time was short and many patients with incomplete data were excluded. Thirdly, radiographs were not standardized, which sometimes made it difficult to measure the angles and slopes appropriately. However, this occurred equally in both groups. Fourthly, no preoperative KSS data were available, so we could not compare preoperative and postoperative data. Fifthly, it was impossible to reliably measure blood loss, because patients were operated with and without tourniquets. The blood loss documented in the operation report was used for this study. Finally, the training level of the residents was not registered, so residents at different stages of their training could have been involved. If these data had been available we could have performed sub-analyses of the different levels of training.

### Future research

In further research, we intend to analyze the process in which residents and supervisors decide who will perform the procedure. We will investigate more patient outcome data related to medical education and training, for example total hip arthroplasty, and we will design a system to use this data in the training and assessment of residents and supervisors.

## Conclusion

Patient outcomes at 1‑year follow-up did not differ between total knee arthroplasties performed by supervised orthopaedic residents or by experienced orthopaedic surgeons concerning complication rate, KSS and implant position 1 year after primary total knee arthroplasty. Supervised orthopaedic residents needed 10 minutes more time to perform a total knee arthroplasty than experienced orthopaedic surgeons, but they performed equally well in terms of KSS, radiographic implant position and complication rate. This finding implies that the current, generally accepted practice of orthopaedic surgical skills training can raise the performance of supervised residents to levels equal to those of experienced orthopaedic surgeons.
